# A Proposal for Automatic Fruit Harvesting by Combining a Low Cost Stereovision Camera and a Robotic Arm

**DOI:** 10.3390/s140711557

**Published:** 2014-06-30

**Authors:** Davinia Font, Tomàs Pallejà, Marcel Tresanchez, David Runcan, Javier Moreno, Dani Martínez, Mercè Teixidó, Jordi Palacín

**Affiliations:** Department of Computer Science and Industrial Engineering, Universitat de Lleida, Jaume II, 69, Lleida 25001, Spain; E-Mails: dfont@diei.udl.cat (D.F.); tpalleja@diei.udl.cat (T.P.); mtresanchez@diei.udl.cat (M.T.); robotica@udl.cat (D.R.); jmoreno@diei.udl.cat (J.M.); dmartinez@diei.udl.cat (D.M.); teixido@diei.udl.cat (M.T.)

**Keywords:** fruit harvesting, stereovision system, size estimate, distance estimate, position estimate, robotic arm

## Abstract

This paper proposes the development of an automatic fruit harvesting system by combining a low cost stereovision camera and a robotic arm placed in the gripper tool. The stereovision camera is used to estimate the size, distance and position of the fruits whereas the robotic arm is used to mechanically pickup the fruits. The low cost stereovision system has been tested in laboratory conditions with a reference small object, an apple and a pear at 10 different intermediate distances from the camera. The average distance error was from 4% to 5%, and the average diameter error was up to 30% in the case of a small object and in a range from 2% to 6% in the case of a pear and an apple. The stereovision system has been attached to the gripper tool in order to obtain relative distance, orientation and size of the fruit. The harvesting stage requires the initial fruit location, the computation of the inverse kinematics of the robotic arm in order to place the gripper tool in front of the fruit, and a final pickup approach by iteratively adjusting the vertical and horizontal position of the gripper tool in a closed visual loop. The complete system has been tested in controlled laboratory conditions with uniform illumination applied to the fruits. As a future work, this system will be tested and improved in conventional outdoor farming conditions.

## 1. Introduction

The agriculture industry is demanding technological solutions focused on automating agriculture tasks in order to increase the production and benefits while reducing time and costs. These technological solutions are mostly based on the application of sensor-based technologies. A comprehensive description can be found in [1] where the most recent research focused on solving agriculture and forestry tasks by using sensors is summarized.

Automation of agriculture tasks has improved all phases of the industrial process, from the pre-harvest, to the harvest and post-harvest stages. For example, in the pre-harvest stage, the application of agro-chemicals in orchards has been automated with the aim of controlling weeds [2] and improving pesticide applications [3]. Canopy characterization [4] greatly contributes to improve agro-chemicals applications. In the case of the harvesting stage, the already proposed systems [5] requires an estimate of the position and size of the fruits in the trees [6–8] and other parameters such as its ripeness stage [8,9]. These estimates can be performed by using a stereo vision system [10]. Finally, in the case of the post-harvesting stages, the most important tasks are related with the estimate of fruit production [11] and quality [8,12] by detecting skin defects [13] or by validating fruit variety [14].

The new contribution of the paper is the combined application of a low cost stereovision system and a low cost robotic arm in order to define an automatic fruit harvesting system. The stereovision is placed in the gripper of the robotic arm in order to detect and locate the fruits in the trees and guide the automatic pickup of the selected fruits. The location of the fruits is performed by matching fruit centroids instead of matching the complete stereo-image as a way to reduce matching complexity algorithm and foster the future development of real-time industrial systems. This paper is focused on the assessment of reference baseline location, guidance and pickup performances in laboratory conditions; future works will optimize and assess the farming operation performances of the complete harvesting system.

## 2. Related Work

The definition of a high quality fresh fruit harvesting system a complex task that requires: the automatic detection of the fruits, the estimate or their size and relative location and orientation, and the definition and control of a non-stressing pickup procedure. The accurate detection of fruits in trees can be addressed with different approaches such as the use of a monocular camera attached to a gripper tool [15] in order to control a mechanical harvesting. In this case, the distance to the fruit was estimated analytically by displacing the camera a known distance and by measuring the fruit radius before and after this displacement. Then, the alignment of the gripper tool with the fruit was performed by matching the center of the fruit with the center of the image. The proposal of [16] also uses a monocular camera and a stepper motor as a displacement device in order to generate depth maps of the scene. In this case, the vision system was designed to reconstruct 3D natural complex scenes. This paper proposes a similar approach based on the use of a low cost stereovision system in a robotic arm in order to estimate the distance to the fruit without having to maneuver the robotic arm to change the angle of view and object perspective.

In the case of using a stereovision system [17], the main problem is to find the correlated information in two images with different views of the same area or object. In most cases, instead of matching pixel by pixel features, the targets are detected on the images and their centroids are used as a landmark in order to estimate the distance of the object. This procedure can be affected by geometric camera nonlinearities such as an offset in the position of the center of the image, skew factor or lens distortion that can be corrected with a specific camera calibration procedure. In [10], the proposal was the implementation of a real-time stereovision system in order to estimate the distance and size of an object. In this proposal, the object was firstly detected in both images and segmented before applying a connected component analysis and a blob extraction technique in order to extract all the information needed: size and distance measurements. This method provides accurate distance and size estimations spending 65 ms in the process. In [18], the effects of using a stereo vision system applied to apple-picking robots were studied under different target circumstances and in a working distance from 300 to 1100 mm. In this case, the first analysis required manual operation and consisted on attaching a small target on the apples whereas the second analysis was performed automatically by computing the centroid of the segmented apples. The error in the distance estimate was 0.63% in the first analysis and 3.54% in the second analysis. In [19], a structured-light stereo vision system was proposed to detect mature tomato by applying a threshold to the Hue and Saturation layers and then a structured light was used to locate its position and size. The ripeness was estimated by analyzing the Cb color layer. The results showed an error in the estimate of the tomato radius less than 5 mm and an error in the distance less than 7 mm.

There are some examples in the literature that apply a stereovision system in the control of a robotized arm, but very few designed for automatic fruit harvesting. For example, in [20], a stereo vision system was integrated in an automatic harvesting system with the aim of locating fruit on a simulated indoor tree and to correct the trajectory performed by a robotic arm in a virtual environment in order to pickup fruits. The conclusion was that the stereo vision system was feasible for positioning fruits and to control robot operation in real-time. Alternatively, in [21] a robot manipulator was proposed for the automatic harvesting of citrus. This paper proposed the development of vision-based estimation and control system for robotic fruit harvesting by analyzing the stability and performance of the closed-loop control system. The control was performed by combining the information provided by a fixed camera and a camera in the hand on the robotic manipulator.

Finally, the specific task of size and distance fruit estimate can be performed with alternative sensing devices. For example, in [22] the proposal was the use of two 2D LIDARs in order to detect position and size of asparagus. In [23], a laser ranging sensor in combination with a machine vision system was used as a real-time fruit detection system achieving results of 100% accuracy when detecting single fruits in different lighting conditions. In this case, the fruit detection system was combined with an effector designed to detach fruits similarly to a human picker achieving an average picking success rate of 90%. In [24], a stereovision system was combined with a projector in order to illuminate the scene with different patterns. In this case, the use of these structured patterns simplified the detection of matching correspondences between the stereo images and improved the procedure for 3D scenario reconstruction.

## 3. Materials

This section describes the image acquisition system used to estimate fruit location, the vision targets used in the experiments, and the mechanical device proposed to pick up the fruits. The control developed to guide the robotic arm in order to harvest fruits is also presented.

### 3.1. Stereovision Image Acquisition System

**Figure 1.**
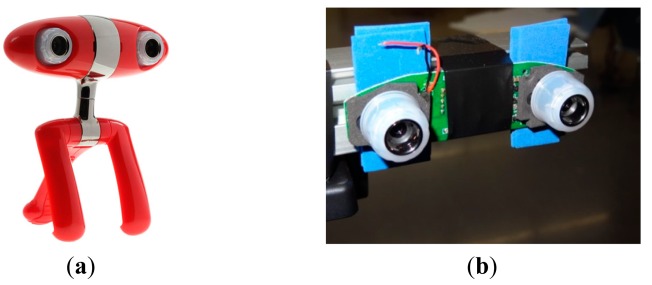
Minoru 3D USB Webcam. (**a**) External view; (**b**) Detail of the two cameras.

**Figure 2.**
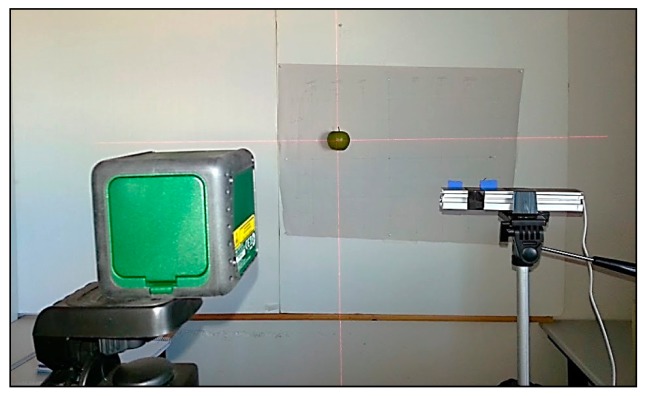
Experimental setup for grid measurement.

The image acquisition device used in this work is a low cost commercial Minoru 3D USB Webcam [25] (Figure 1a). This image acquisition device uses two VGA CMOS color sensors with a resolution of 800 × 600 pixels (Figure 1b). These two cameras are placed in the same plane at a distance of 60 mm from each other. The device can be configured in order to provide two individual images of both cameras or a combined stereo image. In both cases the images are not synchronized and the maximum shutter deviation expected is 16.5 ms. In this paper, the image acquisition device will be used in combination with a red cross laser pointer for accurate target positioning. Figure 2 shows the complete experimental setup. The red cross will be used as a reference in order to place manually the target fruits at exact grid positions and validate their automatically detected positions.

### 3.2. Vision Targets

**Figure 3.**
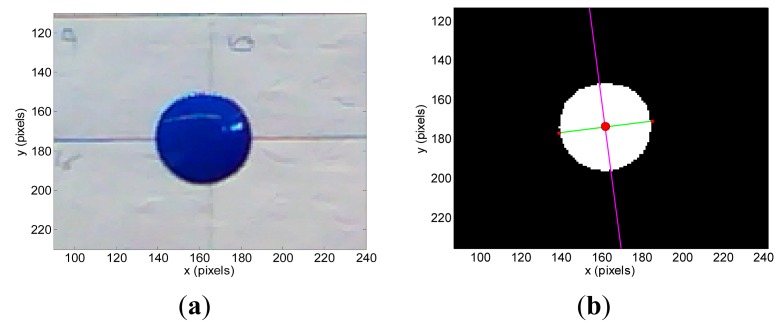
Pushpin: (**a**) original image and (**b**) segmented image showing the centroid (red dot), inclination (magenta line) and diameter in pixels (green line).

**Figure 4.**
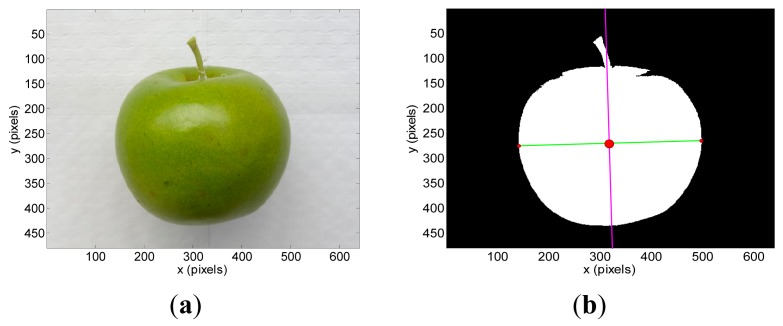
Apple: (**a**) original image and (**b**) segmented image showing the centroid (red dot), inclination (magenta line) and diameter in pixels (green line).

**Figure 5.**
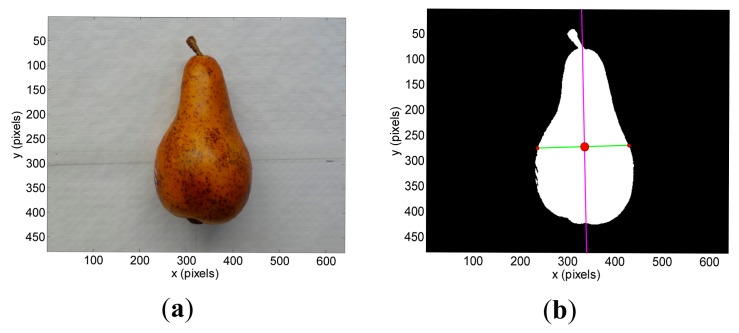
Pear: (**a**) original image and (**b**) segmented image showing the centroid (red dot), inclination (magenta line) and diameter in pixels (green line).

The vision targets tested in the experiments were a blue pushpin that will be used as a reference small and planar object (Figure 3a), a green apple (Figure 4a), and a brown pear (Figure 5a). Table 1 summarizes their sizes and diameters.

### 3.3. Robotic Arm for Fruit Harvesting

**Figure 6.**
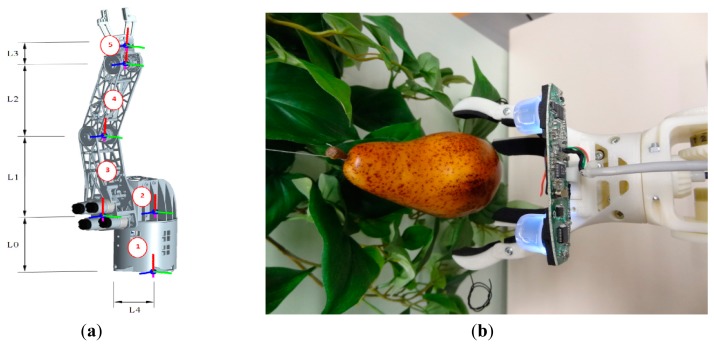
(**a**) Robotic arm design; (**b**) Detail of the gripper tool with the imaging device.

The proposed stereovision system will be applied to control a robotic arm designed for automatic fruit harvesting (Figure 6). The robotic arm has been created with a Dimension SST 1200es 3D rapid prototyping printer in ABS (FullCure720) plastic material which includes six low cost DC gear motors controlled by a Cortex-M4F ARM STM32F407VGT6 microcontroller that provides velocity and speed control and different connectivity options. The robotic arm is composed by five linked members and a manually interchangeable gripper (see Figure 6a). In this paper the initial position of the robotic arm has components 3, 4 and 5 (labeled in red color) vertically aligned. In the final application the robotic arm will be attached to a harvesting platform in order to automatically pickup the fruits from the trees. The base of the robotic arm (Figure 6a, component 2) is able to spin 360° on its x-axis (red line) and place the gripper in the adequate radial position for fruit harvesting. Then, components 3 and 4 can spin 260° (130° on each side from the original position) on their z-axis (blue line) in order to approximate the robotic gripper to the fruit. Finally, member (5) has two degrees of freedom being able to spin 260° (130° on each side from the original position) on its z-axis (blue line) and 360° on its x-axis (red line) giving the two specified motions to the robotic gripper. Table 2 summarizes the dimensions of the main components of the robotic arm.

The proposed design of the gripper tool is based on the use of two upper moving fingers to grab the fruit and two lower fixed fingers to hold the fruit (Figure 6b). The lower holding fixed fingers minimize the pressure required to grab the fruit with the moving fingers and contributes to reduce the mechanical stress of the fruit pickup procedure. This design was inspired in the mechanical action performed by a human hand during the process of holding and picking up fruits. The gripper tool uses a single DC motor for opening and closing the moving fingers which are normally open. This system is very sensitive; the closing (or fruit grabbing) procedure is stopped when the torque applied by the motor of the fingers increases more than 10%. The torque applied by the DC motor is estimated by measuring its current. Additionally, the contact surfaces of the gripper tool have a soft foam rubber to reduce the local pressure applied to the fruit. Depending on the results obtained in future usage tests the gripper tool can be improved with a robust adaptive impedance control [26] or with more degrees of freedom in order to obtain information of the shape of the fruits [27].

### 3.4. Guidance of the Robotic Arm

The guidance of the robotic arm was addressed by computing the inverse kinematics of the robotic arm which, in this case, can be performed analytically by simplifying the complete system as a two-joint robotic arm. Under this simplification, only two absolute angles are truly needed in order to place the tip of the robotic arm in a desired position. The first value defines the angle between the components 2 and 3 whereas the second value defines the angle between the components 3 and 4 of the robotic arm. This simplified computation requires two assumptions: (1) the rotation of the component 2 around its x-axis (red line) can be performed independently from the other joints until the robotic arm reaches an optimal radial orientation to the current selected fruit; (2) the optimal orientation of the gripper in order to pick up the fruits is always parallel to the ground.

In this paper, the simulation and validation of the guidance of the robotic arm has been performed by defining a simplified Denavit-Hartenberg (D-H) parametric model [28]. This simplified model represents the relative motion between articulations by using four basic transformations: two translations, “*d*” and “*a*” parameters, (which coincide with the dimension of the components of the robotic arm) and two revolutions, α and θ parameters, defined along the x (red line in Figure 6a) and z (blue line in Figure 6a) axes. The value of these parameters depends on the initial orientation of the robotic arm and on the definition of the coordinate axis which, in this case, is located in the base of the robot arm (Figure 6a, component 1). Table 3 shows the initial position and motion range of the α and θ parameters whereas Table 4 represents the complete simplified D-H parametric model of the robotic arm that can be used to compute the final position (x, y, z spatial coordinates) of the gripper of robotic arm.

## 4. Stereovision Fruit Detection Accuracy

The control of the robotic arm requires an estimate of the fruit distances, positions and sizes in the trees in order to propose an automatic fruit harvesting procedure. In this paper, this estimate will be performed with a stereovision system.

### 4.1. Experimental Setup

**Figure 7.**
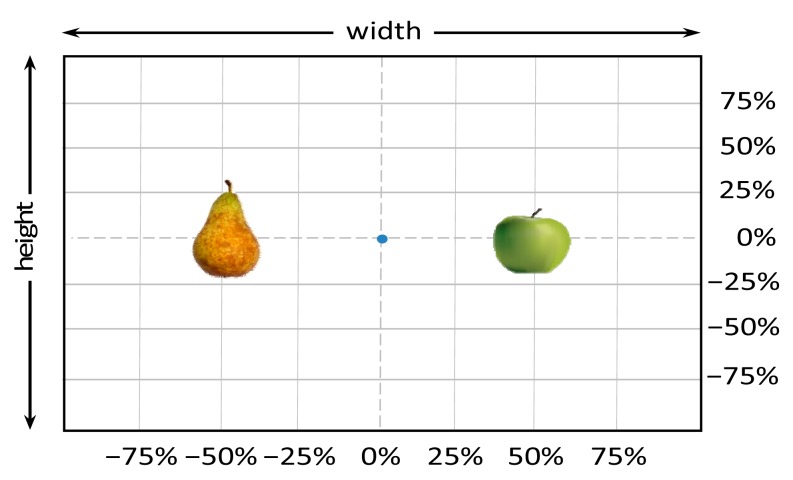
Relative grid definition and targets used in the experimental setup.

Figure 7 shows the experimental setup used in this paper in order to estimate the detection accuracy of a low cost stereovision system in the case of detecting three different targets: a blue pushpin, a green apple, and a brown pear. This experimental setup will be used to obtain 49 images (in the intersection of a 7 × 7 relative grid) per target and distance, covering a total of 1470 stereo images in a distance range from 200 mm up to 2000 mm in steps of 200 mm.

**Figure 8.**
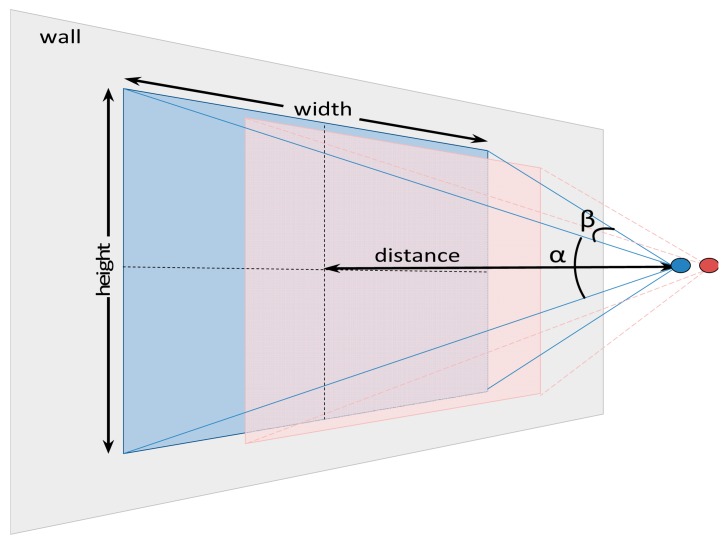
Representation of the right (red point) and left camera (blue point) and their visible area.

**Figure 9.**
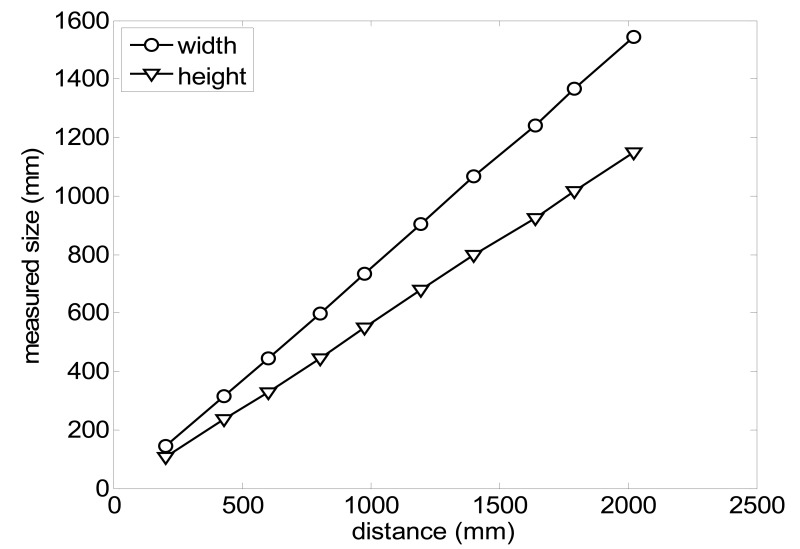
Grid size in function of the distance between the camera and the target.

The size (width and height) of the grid is always the visible area of the left camera (see Figure 8) and this area depends on the distance between the camera and the targets. Figure 9 shows the relationship between the size and the distance which can be used to estimate the horizontal (39.68°) and vertical (30.06°) focal angles of the cameras of the stereovision system.

### 4.2. Image Processing: Target Centroid, Inclination and Diameter Estimate

The image processing stage involves background segmentation and the estimate, for the different targets proposed, of the inclination in grades and the centroid and diameter in pixels. The white background used in the experimental setup simplifies the procedure of detecting the background in both RGB color images obtained with the stereovision system. In this laboratory case, a pixel is classified as a member of the background if their individual RGB color intensities are all greater than 0.8.

The segmented images used to have isolated noisy background pixels that can be removed from the images by applying morphological operators or a hole filling algorithm. Then, the region covered by the target object is the remaining non-background area of the image. This unique and well defined region in the images allows the computation of the position of the centroid (*x_c_*, *y_c_*) (center of mass of the region), inclination *ω* (angle between the x-axis and the major axis of the ellipse that has the same second-moments as the region), and diameter *Ф_p_* (length of the minor axis of the ellipse that has the same normalized second central moments as the region). The computation of the centroid is required for both images obtained with the stereovision system whereas the estimate of the inclination and diameter can be limited to one image. Figures 3a, 4a and 5a show the detail of the targets analyzed in one example image while Figures 3b, 4b and 5b show the detection results: centroid location (red dot), inclination (magenta line) and diameter (green line).

### 4.3. Distance, Position and Diameter Estimate

**Figure 10.**
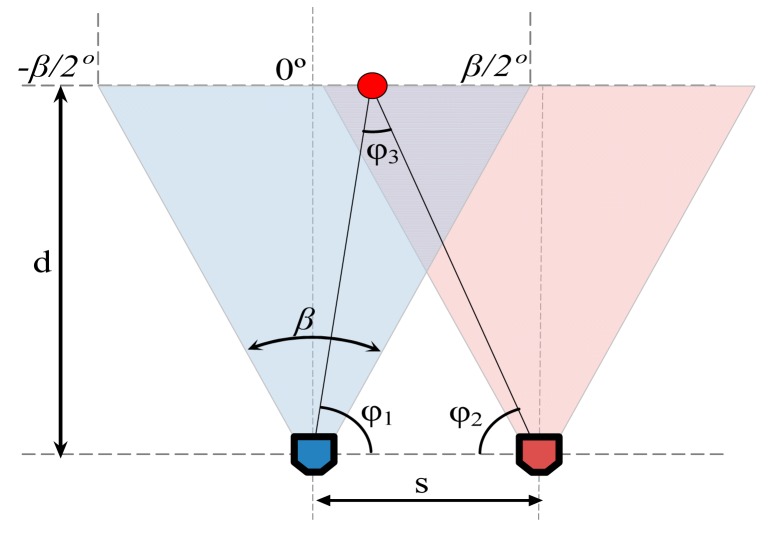
Schematic representation of the parameters involved in a distance estimate with a stereovision system.

The acquisition of two stereovision images showing the same object from different and known point of views allows the analytic estimate of the target relative distance, relative position and absolute diameter. Figure 10 shows a schematic representation of the parameters involved in the estimate of the distance where *s* is the distance between cameras, and *β* is the horizontal angle of view of the cameras.

These parameters allows the estimate of the distance to a pixel located in the column *x_1_* of the left image and *x_2_* of the right image by analytically computing the angles *φ_1_*, *φ_2_* and *φ_3_*. Then, the distance *d* from the planes of the two stereo cameras to the plane of the pixel can be obtained with:

(1) $$d=\left|\frac{s\cdot \sin\varphi_{1}\cdot \sin\varphi_{2}}{\sin\varphi_{3}}\right|$$

The procedure for distance estimate can be improved by correcting the geometric camera nonlinearities with a specific camera calibration procedure [16,29]. Table 5 shows the intrinsic and extrinsic camera calibration parameters found for the low cost stereovision system used in this paper.

The relative position of a pixel (*x*, *y*) can be computed from the distance *d*, the size of the image (*rows*, *cols*), and the relative location of the pixel in one image (*x_1_*, *y_1_*) of the stereovision although this computation requires the determination of the scale (*xScale*, *yScale*) of the pixels in the image:

(2) $$\text{xScale}=\frac{2d\cdot \tan\frac{\beta }{2}}{\text{cols}},\;\text{yScale}=\frac{2d\cdot \tan\frac{\alpha }{2}}{\text{rows}}$$

(3) $$x=\left(x_{1}-\frac{\text{cols}}{2}\right)\cdot \text{xScale},\;y=\left(y_{1}-\frac{\text{rows}}{2}\right)\cdot \text{yScale},\;y_{o}=\left(-y_{1}+\frac{\text{row}}{2}\right)\text{yRel}$$

Finally, the real target diameter *Ф* expressed in millimeters can be computed analytically from the apparent diameter in pixels *Ф_1_* obtained from the left image of the stereovision system:

(4) $$\Phi =\sqrt{{\left(\Phi_{1}\cdot \cos\omega \cdot \text{xScale}\right)}^{2}+{\left(\Phi_{1}\cdot \sin\omega \cdot \text{yScale}\right)}^{2}}$$

### 4.4. Experimental Results

Table 6 summarizes the average (AV) and standard deviation (SD) error values obtained during the estimate of the distance, position and diameter of the targets with the stereovision system. Table 6 shows one column with the results obtained when processing the information from the raw images (camera not calibrated) and another column with the results obtained when the information from the images was geometrically corrected (calibrated camera). Complementarily, Table 7 shows the distance error, the position error, and the diameter error in the case of using a calibrated camera when placing an apple in the 49 different grid locations previously defined. The information of Table 7 is summarized in an average form in Table 6. For example, in the case of estimating the error at 977 mm when using the camera calibrated method, the average distance error obtained in the 49 images analyzed (the apple was placed at 49 grid locations) was 21.16 mm and the standard deviation 4.29 mm; the average position error was 6.98 mm and the standard deviation 3.91; and the average diameter error was 1.55 mm and the standard deviation 0.98 mm.

Table 6 shows that the absolute average and standard deviation obtained improves largely in the case of correcting the geometric distortion of the cameras. In the case of using a pushpin as a vision target with the camera calibrated, the average distance error was approximately 5% in a range from 203 to 2015 mm but the average error in the estimate of the diameter was in a range 12% to 30% because of its small size (the centroid of the pushpin is computed with very few pixels). In the case of the apple target, the average distance error was approximately 4% and the average diameter error was in a range from 2% to 4%. Finally, in the case of the pear, the average distance error was approximately 4% and the average diameter error was in a range from 4% to 6%. These results validate the use of the proposed low cost stereovision image acquisition system for different targets and increases the fruit distance range analyzed previously in [20] from 850 mm to 2025 mm.

## 5. Automatic Harvesting

The complete proposal of an automatic fruit harvesting system requires the control of the robotic arm based on the positioning information provided by the stereovision system. The stereovision system is directly attached to the gripper of the robotic arm (Figure 6b) in order to obtain relative positioning information between the gripper tool and the fruit. The complete development of this experimental assessment requires four stages: (1) initial fruit detection; (2) rough approach to a selected fruit; (3) fine approach to a selected fruit; and (4) fruit pickup. In this paper, the automatic fruit harvesting system has been applied to pick up some pears in controlled laboratory conditions. In the future, this harvesting system will be validated in real outdoor farming conditions.

### 5.1. Initial Fruit Detection

The initial fruit detection procedure, limited to the case of harvesting pears and performed in laboratory conditions, has been primarily addressed by applying a simple RGB color threshold [20] to the stereovision images but real outdoor conditions affected by changing illumination conditions may require a more elaborated segmentation procedure.

**Figure 11.**
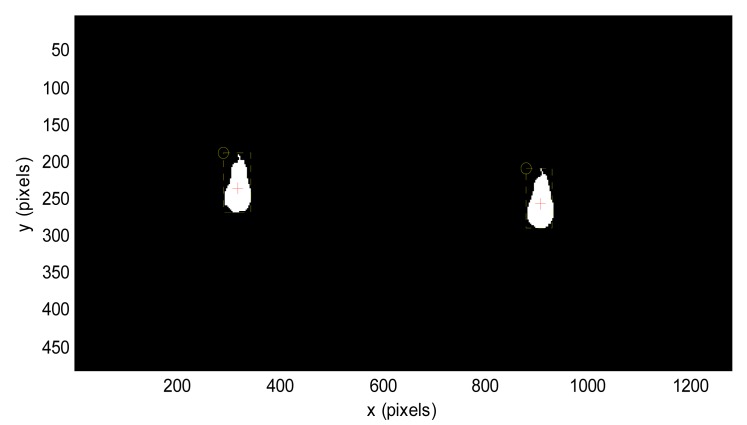
Example of fruit segmentation and location in a stereovision image.

The assumptions made in this initial fruit detection were: (1) the stereovision system, placed in the gripper tool of the robotic arm, will be always in a known initial reference position; and (2) the distance range of the fruits will be from 203 to 2025 mm from the stereovision system. Then, the stereovision system can provide an estimate of the distance, location and diameter of the fruit, affected by the detection uncertainty stated in Table 6. This procedure ends with the selection of a fruit in the image based on their diameter estimate. Figure 11 shows the fruit segmentation results obtained in the case of detecting a pear in laboratory conditions.

### 5.2. Rough Approach to a Fruit

**Figure 12.**
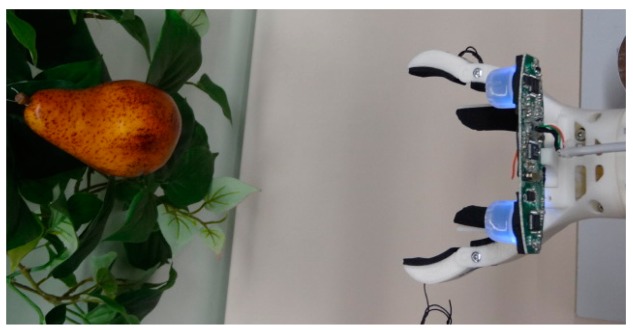
Example of a rough approach to a fruit.

The initial displacements of the robotic arm in order to move the gripper tool in the direction of a selected fruit must be considered as a rough approach that will be affected by the uncertainty of the detection procedure. The estimate of the distance and position of a selected fruit relative to the stereovision system located in the gripper tool is first computed in order to rotate the robotic arm in the direction of the fruit. The results of Table 7 showed that the distance, position and diameter errors used to be lower when the fruit was placed in the center of the image. So the estimate of the distance to the fruit is obtained again and used to compute the inverse kinematics of the robotic arm in order to move the gripper tool very close to the selected fruit (at an approximate distance of 250 mm). Figure 12 shows an image of the result of this rough approach stage.

### 5.3. Fine Approach to a Fruit

**Figure 13.**
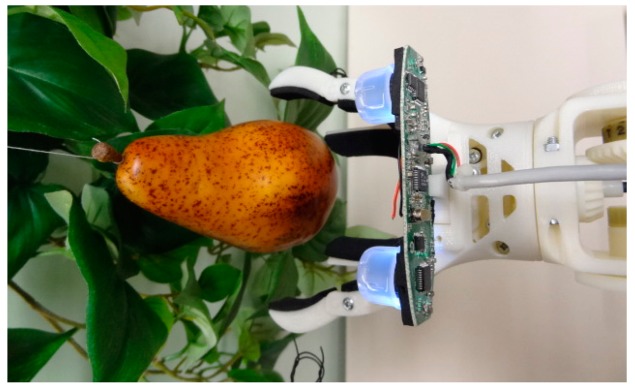
Example of a fine approach to a fruit.

A specific procedure is proposed in order to control the fine displacement of the gripper tool in order to pick up a selected fruit. Like in [15], this fine approach is based on moving forward the gripper tool of the robotic arm according to the position of the centroid of the selected fruit in the image acquired by the stereovision system. Then this fine approach algorithm suggests small vertical and horizontal relative displacements the gripper tool in order to center and finally pickup the fruit. Figure 13 shows an image of the result of this fine approach.

**Figure 14.**
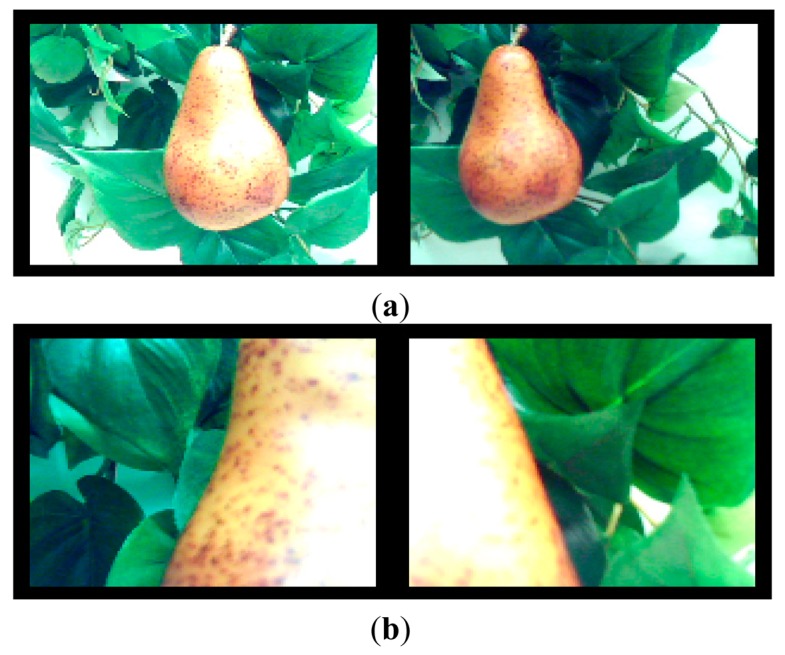
Example of stereovision images obtained while performing a fine approach to a fruit: (**a**) at the beginning of the approach; and (**b**) at the end of the approach.

The use of the proposed stereovision system in this fine approach is somewhat problematic because the limited angle of view of the stereovision system does not provide a complete image of the tracked fruit at very short distances. In order to illustrate this problem, Figure 14a shows an image of a fruit in front of the gripper before starting the fine approach and Figure 14b shows the image obtained when the gripper tool was ready to pick up the fruit (position shown in Figure 13). The problem is then to stop this fine displacement in order to pick up properly the fruit with the gripper tool. In this paper, this iterative procedure was stopped by applying a threshold to the area of the fruit in the proximity images (Figure 14a). However, this estimate may require the use of an additional contact or non-contact sensor in the gripper tool in order to stop this iterative fine approach when picking different types of fruits.

### 5.4. Fruit Pickup

**Figure 15.**
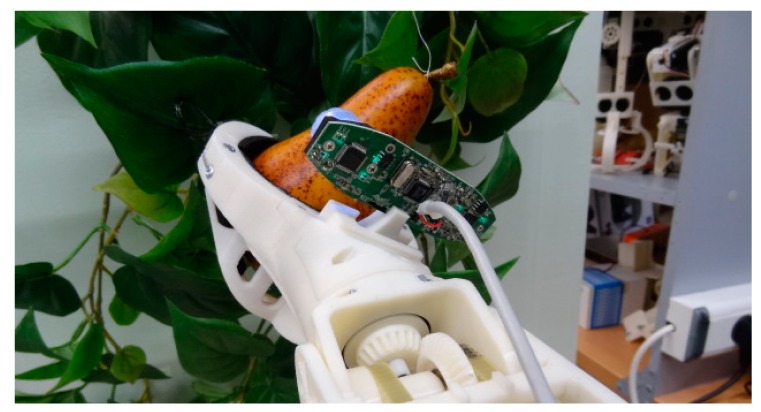
Example of fruit pickup in laboratory conditions.

Finally, the mechanical actions proposed to pick up a pear are: (1) close the gripper and (2) rotate the gripper in order to simulate the motion of the hand performed by a human operator during a pickup fruit operation. Figure 15 shows an image of the resulting mechanical action. With such approach, the effective pressure applied to the fruit is very week as the role of the moving fingers is just to avoid lateral fruit displacement instead of holding the fruit in the air, task performed with the lower fixed fingers. Future works will be focused on analyzing the effective pressure applied by the gripper tool and by verifying the effect of the proposed rotation of the gripper in pears and in other fruits.

### 5.5. Fruit Pickup Performances

The proposed automatic harvesting system has been tested in laboratory conditions. Tables 8 and 9 show some detection results obtained in the initial fruit detection procedure which is the most critical stage of the complete harvesting procedure. For the sake of comparison, the images analyzed have been segmented by applying a color intensity threshold (Table 8) and by applying a detection based on the definition of Linear Color Models (LCM) [7] (Table 9).

Table 8 shows different cases of images obtained with the stereovision system and the segmentation results obtained by applying the Otsu threshold segmentation [30] combined with object size filtering (objects with less than 200 connected pixels are discarded) for noise reduction, and a final object filling just for better representation. In general, the differentiation between the reddish pears and the greenish foliage is not problematic but the inclusion of an occluding brownish synthetic branch in front of the pear is not correctly detected by this basic segmentation procedure. In this case, the inclusion of additional morphological conditions such as the verification of the angle of orientation (discarded if lower than 45°), diameter and axial symmetry enables a preventive discarding of the current pear as candidate for automatic harvesting although the selection still fails in some cases.

Alternatively, Table 9 shows the segmentation results obtained by LCM segmentation method which is robust to illumination changes and texture color similitude. This segmentation method is applied to the same images shown previously in Table 8 in order to compare the results. In this case, the pixels of the synthetic and overlapping brownish branch are not classified as members of the pear class and the pear analyzed appear divided in different parts, losing the size and axial symmetry of a typical pear. In general, the occlusion of the fruit by branches is a problematic harvesting case that must be detected and avoided. The occlusion of the fruit by leaves may require the injection of some air over the surface of the fruit in order to re-detect the fruit and re-evaluate the remaining fruit overlapping remains and the harvesting procedure avoided.

Finally, Table 10 summarized the fruit pickup-time performances obtained with an Intel i7 computer for the different algorithms and steps involved in the complete harvesting procedure. The main time-limitation was imposed by the image acquisition system which provides images at a continuous and unsupervised frame rate of 25 frames per seconds but with only an effective lapse of 30 ms between images. This means that, after stereovision image acquisition, the image processing algorithms have less than 30 ms in order to operate at full camera frame rate and avoid image skipping.

Table 10 shows that the fruit detection stage in the stereo image acquired, composed by intensity color segmentation, image labeling and centroid fruit estimate required less than 30 ms. The computation of the inverse kinematics was also very fast because it was based on a deterministic computation without any iterative procedure. The rough approach stage is very dependent on the mechanical design, motors and configuration of the robotic arm; in the current prototype this initial approach required 4.2 s in average although this value can be reduced easily just by reducing the gear of the DC motors used in the robotic arm. The fine approach to a fruit is currently performed in an iterative way by using the information of the image acquisition system in a visual control loop. This iterative implementation is not optimal as it spends 7.9 s in average in a short approach but is proposed as it is able to automatically compensate any lateral displacement of the robotic arm when carried in a harvesting platform. The mechanical action required to pick up the fruit is currently configured as a fixed displacement and requires approximately 2 s. In average, the proposed automatic system for fruit harvesting is able to pick up one pear from the tree in an average time of 16 s in the case of laboratory conditions. Future work will be focused on evaluating fruit pickup system performances in a real farming operation and in optimizing the proposed automatic fruit harvesting prototype.

## 6. Conclusions and Future Work

This paper proposes the development of a low cost fruit harvesting system by combining a low cost stereovision system and a robotic arm. The stereovision system, placed in the gripper tool, will provide direct information and control of the actions performed by the robotic arm. The paper first proposes the estimation of fruit target distance, position and size accuracy when using a low cost stereovision system and in the cases of correcting and not correcting geometric camera distortions. A total of 1470 images have been processed corresponding to three targets: a reference small pushpin, an apple and a pear; these targets were located in 49 positions of a relative grid, and in 10 intermediate distances from 205 to 2050 mm. In all cases, the distance, position and size error was lower in the case of correcting the geometric distortions originated by the cameras; obtaining average distance errors in a range from 4% to 5% in the case of a pushpin as a target and in a range from 2% to 6% in the case of a pear and an apple as targets. These results validate the use of the proposed low cost stereovision system for fruit distance and parameter estimate.

Then, the paper proposes the complete development of a fruit harvesting system based on the use of a stereovision system attached into the gripper tool of the robotic arm. The gripper tool has been designed to facilitate fruit holding and manipulation whereas the stereovision system provides fruit size and positioning information relative to the gripper tool. The complete automatic fruit harvesting procedure was performed by developing four intermediate stages: (1) initial fruit detection; (2) rough approach to a selected fruit; (3) fine approach to a selected fruit; and (4) fruit pickup.

The initial fruit detection stage was specifically tested with two segmentation algorithms in the case of using reddish pears as fruit targets. The time-performances of the complete harvesting prototype was also tested, requiring an average time of 16 s to detect and pick up a pear whereas the 95% of this time was originated in mechanical limitations imposed to the robotic arm. As a future work, this harvesting system will be validated and optimized in real outdoor farming conditions. The final goal will be the combination of several robotic arms operating in parallel in order to define a versatile robotized harvesting platform.

## Figures and Tables

**Table 1.** Vision target sizes.

	Width (mm)	High (mm)	Diameter (mm)
Pushpin	10.5	10.5	10.5
Apple	80.5	64.9	80.5
Pear	67.6	110.9	61.3

**Table 2.** Robotic arm dimensions.

Parameter	Length (mm)
L0	160
L1	140
L2	200
L3	80
L4	90

**Table 3.** Ranges of the parameters α and theta θ of the joints.

Parameter	Initial Position	Maximal Position	Minimal Position	Parameter	Initial Position	Maximal Position	Minimal Position
α_1_	0°	0°	0°	θ_1_	0°	0°	0°
α_2_	0°	−180°	180°	θ_2_	0°	0°	0°
α_3_	0°	0°	0°	θ_3_	0°	0°	0°
α_4_	0°	0°	0°	θ_4_	0°	−130°	130°
α_5_	0°	0°	0°	θ_5_	0°	−130°	130°
α_6_	0°	0°	0°	θ_6_	0°	−130°	130°
α_7_	90°	−180°	180°	θ_7_	0°	0°	0°

**Table 4.** Fixed D-H parameters for the robotic arm.

Joint	d	a	α°	θ°
1	0	0	0	0
2	0	L0	α 2	0
3	L4	0	0	0
4	0	L1	0	θ 4
5	0	L2	0	θ 5
6	0	L3	0	θ 6
7	0	0	α 7	0

**Table 5.** Camera calibration parameters.

**Intrinsic**	Right camera	Focal length	fc_r	[846.02 841.61]
		Principal point	cc_r	[327.45 218.01]
		Skew	alpha_r	0.00
		Distortion	kc_r	[−0.13106 0.09150 −0.00076 0.00250 0.00000]

	Left camera	Focal length	fc_l	[865.54 860.67]
		Principal point	cc_l	[309.90 238.60]
		Skew	alpha_l	0.00
		Distortion	kc_l	[−0.11702 −0.00767 0.00116 0.00018 0.00000]

**Extrinsic**	Position of the right camera in relation to the left camera	Rotation	om	[−0.00426 −0.00369 −0.00061]
		Translation	T	[0.06060 −0.00025 −0.00028]

**Table 6.** Error detection in terms of average (AV) and standard deviation (SD).

Target	Distance (mm)	Camera not Calibrated {AV/SD} (mm)			Camera Calibrated {AV/SD} (mm)		

		Distance Error	Position Error	Diameter Error	Distance Error	Position Error	Diameter Error
**Pushpin**	**203**	7.43/1.87	3.18/1.37	0.32/0.22	2.88/1.00	1.30/0.73	0.28/0.19
	**431**	36.13/15.64	5.17/2.67	1.22/1.10	19.02/1.91	2.31/1.33	1.24/1.23
	**605**	74.22/21.19	10.95/6.29	0.69/0.56	48.46/8.75	5.64/2.81	0.75/0.66
	**804**	57.51/30.62	16.87/8.77	1.13/0.83	22.82/25.28	9.79/5.29	1.02/0.73
	**977**	78.06/44.18	18.42/13.83	1.73/1.25	30.32/6.67	3.09/1.75	1.55/1.03
	**1197**	113.17/67.80	31.74/24.66	3.40/2.51	41.10/10.75	9.23/13.29	3.21/2.64
	**1402**	148.70/95.42	37.37/28.53	3.46/2.16	65.40/17.76	3.42/2.51	3.22/2.15
	**1607**	206.40/133.99	48.56/37.67	2.19/1.60	89.30/23.63	5.40/3.67	1.78/1.33
	**1793**	242.12/154.97	58.35/46.43	1.92/1.33	91.96/25.43	5.40/4.28	1.51/0.98
	**2025**	305.05/210.39	79.22/64.39	2.85/1.67	116.03/31.82	8.83/7.61	2.34/1.52

**Apple**	**203**	-	-	-	-	-	-
	**431**	6.92/6.13	8.40/2.26	5.70/1.38	30.11/1.73	6.55/1.86	0.92/0.57
	**605**	28.87/16.35	11.57/6.09	8.44/3.68	5.82/3.48	7.94/2.65	3.60/1.81
	**804**	40.38/22.85	16.84/9.70	6.05/4.96	29.26/8.90	10.90/8.49	2.71/2.48
	**977**	54.55/30.61	15.93/8.89	5.61/3.28	21.16/4.29	6.98/3.91	1.55/0.98
	**1197**	90.32/51.95	25.14/18.99	6.77/5.21	9.52/ 7.37	8.17/9.38	1.45/2.62
	**1402**	133.77/80.46	35.19/23.31	12.30/8.77	20.10/15.14	10.02/5.30	5.98/4.15
	**1607**	197.87/113.40	51.60/38.54	10.70/6.99	42.74/16.56	9.50/5.88	2.23/2.14
	**1793**	231.27/134.93	59.21/45.40	10.94/6.86	39.26/15.78	7.65/4.75	1.48/1.51
	**2025**	288.93/174.68	75.99/60.56	13.00/8.85	65.14/24.93	9.94/5.17	2.80/2.50

**Pear**	**203**	-	-	-	-	-	-
	**431**	7.97/4.59	11.95/1.97	5.73/1.41	19.15/1.18	10.62/1.93	2.76/1.01
	**605**	33.60/19.08	14.45/5.73	7.91/2.68	5.76/5.03	9.66/2.32	4.99/1.13
	**804**	38.71/21.54	23.74/5.56	4.62/3.02	22.02/5.24	19.76/3.91	1.56/1.14
	**977**	55.51/28.70	18.92/7.75	4.78/2.87	12.46/2.90	10.48/4.13	1.36/0.74
	**1197**	94.37/54.18	27.57/15.66	6.54/4.26	7.03/7.04	10.98/4.17	2.73/1.35
	**1402**	148.79/82.45	43.37/29.50	9.57/6.53	19.44/16.06	13.41/4.31	4.07/3.50
	**1607**	196.39/116.06	54.22/37.79	8.27/5.43	48.55/16.43	14.12/5.53	2.73/1.72
	**1793**	230.41/134.35	62.43/45.09	8.71/5.35	47.21/13.27	10.57/3.18	2.86/1.48
	**2025**	298.65/183.36	81.01/62.16	10.83/7.29	76.26/20.67	13.38/6.30	3.80/2.32

**Table 7.** Distribution of the errors in the measurement grid in case of detecting an apple with a calibrated camera.

Distance (mm)	Distance Error (mm)	Position Error (mm)	Diameter Error (mm)
**977**	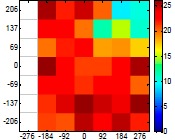	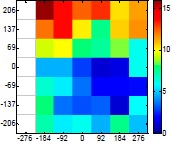	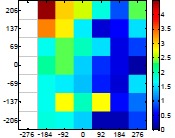
**1197**	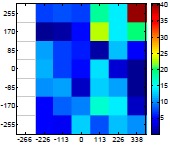	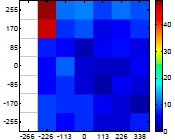	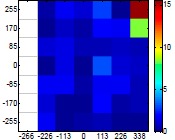
**1402**	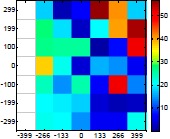	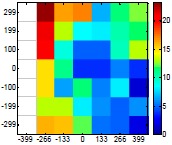	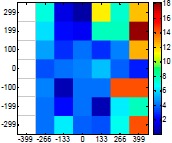
**1607**	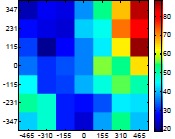	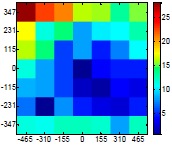	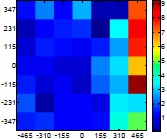
**1793**	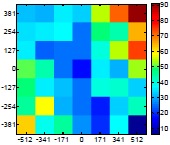	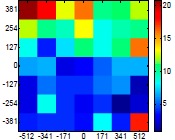	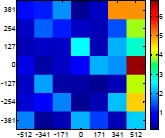
**2025**	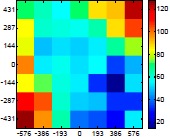	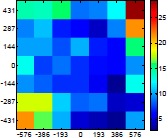	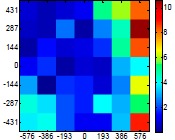

**Table 8.** Fruit detection: Threshold segmentation case.

One Side Image	Segmented Image	Pear Estimate		Candidate for Harvesting

		Diameter	Orientation	
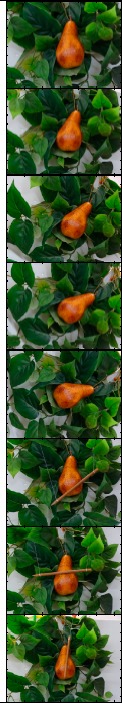	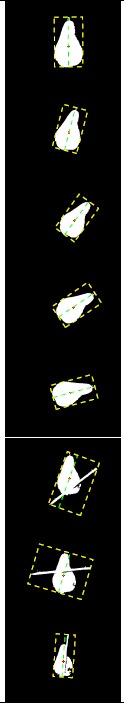	276 pix	89.9°	Yes
		244 pix	75.4°	Yes
		226 pix	53.9°	Yes
		239 pix	33.7°	No (orientation)
		239 pix	14.9°	No (orientation)
		306 pix	70.3°	No (non pear symmetry)
		228 pix	73.4°	No (non pear symmetry)
		236 pix	86.0°	Yes (failed)

**Table 9.** Fruit detection: LCM segmentation [7] case.

One Side Image	Segmented Image	Pear Estimate		Candidate for Harvesting

		Diameter	Orientation	
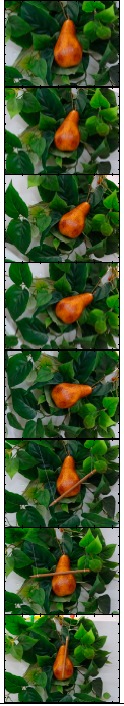	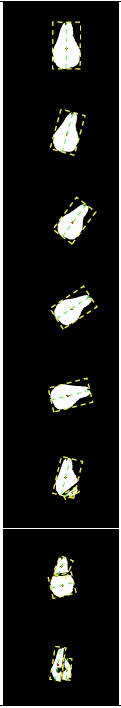	255 pix	89.5°	Yes
		227 pix	74.6°	Yes
		218 pix	53,1°	Yes
		222 pix	32.9°	No (orientation)
		221 pix	13.4°	No (orientation)
		211 pix 87 pix	76.5° 29.2°	No (occluded)
		105 pix 130 pix	67.9° 13.4°	No (overlapping and symmetry)
		117 pix 183 pix	86.4° 83.4°	No (overlapping and symmetry)

**Table 10.** Fruit pickup time-performances.

Fruit Pickup Stage	Algorithm Computation			Robotic Arm Motion		

	min	average	max	min	average	max
Fruit detection		<30 ms				
Inverse kinetatics			5 ms			
Rough approach				3.1 s	4.2 s	9.6 s
Fine approach to a fruit (1 iteration)		<30 ms	35 ms	0.9 s	1.1 s	1.4 s
Fine approach to a fruit (operation complete)					7.9 s	11 s
Fruit pickup					2.0 s	
